# Cdk5 Is Essential for Amphetamine to Increase Dendritic Spine Density in Hippocampal Pyramidal Neurons

**DOI:** 10.3389/fncel.2017.00372

**Published:** 2017-11-24

**Authors:** Soledad Ferreras, Guillermo Fernández, Víctor Danelon, María V. Pisano, Luján Masseroni, Christopher A. Chapleau, Favio A. Krapacher, Estela C. Mlewski, Daniel H. Mascó, Carlos Arias, Lucas Pozzo-Miller, María G. Paglini

**Affiliations:** ^1^Laboratory of Neurophysiology, Instituto de Investigación Médica Mercedes y Martín Ferreyra, INIMEC-CONICET, Universidad Nacional de Córdoba, Córdoba, Argentina; ^2^Department of Neurobiology, Civitan International Research Center, University of Alabama at Birmingham, Birmingham, AL, United States; ^3^Centro de Biología Celular y Molecular, Facultad de Ciencias Exactas, Físicas y Naturales, Universidad Nacional de Córdoba, IIBYT-CONICET, Córdoba, Argentina; ^4^Laboratory of Neurobiology, Instituto de Investigación Médica Mercedes y Martín Ferreyra, INIMEC-CONICET, Universidad Nacional de Córdoba, Córdoba, Argentina; ^5^Virology Institute “Dr. J. M. Vanella”, Facultad de Ciencias Médicas, Universidad Nacional de Córdoba, Córdoba, Argentina

**Keywords:** amphetamine, hippocampus, dendritic spines, Cdk5/p25, calpain, organotypic slice cultures

## Abstract

Psychostimulant drugs of abuse increase dendritic spine density in reward centers of the brain. However, little is known about their effects in the hippocampus, where activity-dependent changes in the density of dendritic spine are associated with learning and memory. Recent reports suggest that Cdk5 plays an important role in drug addiction, but its role in psychostimulant’s effects on dendritic spines in hippocampus remain unknown. We used *in vivo* and *in vitro* approaches to demonstrate that amphetamine increases dendritic spine density in pyramidal neurons of the hippocampus. Primary cultures and organotypic slice cultures were used for cellular, molecular, pharmacological and biochemical analyses of the role of Cdk5/p25 in amphetamine-induced dendritic spine formation. Amphetamine (two-injection protocol) increased dendritic spine density in hippocampal neurons of thy1-green fluorescent protein (GFP) mice, as well as in hippocampal cultured neurons and organotypic slice cultures. Either genetic or pharmacological inhibition of Cdk5 activity prevented the amphetamine–induced increase in dendritic spine density. Amphetamine also increased spine density in neurons overexpressing the strong Cdk5 activator p25. Finally, inhibition of calpain, the protease necessary for the conversion of p35 to p25, prevented amphetamine’s effect on dendritic spine density. We demonstrate, for the first time, that amphetamine increases the density of dendritic spine in hippocampal pyramidal neurons *in vivo* and *in vitro*. Moreover, we show that the Cdk5/p25 signaling and calpain activity are both necessary for the effect of amphetamine on dendritic spine density. The identification of molecular mechanisms underlying psychostimulant effects provides novel and promising therapeutic approaches for the treatment of drug addiction.

## Introduction

Changes in behavior that occur as a function of experience, like environmental enrichment (Engmann et al., 2015), drug addition (Robinson and Kolb, 1997; Rasakham et al., 2014), injuries (Tseng and Hu, 1996; Chen et al., 2003) and throughout aging (Scheibel et al., 1975; Wang et al., 2009), have their basis in the reorganization of excitatory synaptic connections, also known as structural plasticity of dendritic spines. Structural plasticity involves a variety of proteins controlling the organization of the actin cytoskeleton (Schubert and Dotti, 2007; Cingolani and Goda, 2008) and changes in dendritic spine shape, size and number are determined by local actin dynamics (Fischer et al., 2000; Tada and Sheng, 2006; Lai and Ip, 2013). Activity-dependent dynamic changes in spines of hippocampal pyramidal neurons are essential for learning and memory (Yuste and Bonhoeffer, 2001; Kasai et al., 2003).

The Cdk5/p35 signaling complex has been involved in multiple processes during typical brain development, including neuronal migration and the formation of axons and dendrites (Nikolic et al., 1996; Chae et al., 1997; Paglini et al., 1998, 2001; Kwon et al., 1999; Smith and Tsai, 2002), as well as in associative learning and memory in adult rodents (Fischer et al., 2002, 2005; Angelo et al., 2006; Bignante et al., 2008). On the other hand, Cdk5 plays a critical role in the pathophysiology of several neurodegenerative disorders. Deregulation of Cdk5 results in neuronal loss in Parkinson’s, Alzheimer’s and Huntington’s diseases (Cheung and Ip, 2012; Shah and Lahiri, 2014), which underscores the importance of the complex regulation of Cdk5 activity necessary to maintain its activity within physiological levels. Cdk5 kinase activity is regulated in many different ways. The main regulators of its kinase activity are the activators p35 and p39, which are highly expressed in adult brain (Tsai et al., 1994; Tang et al., 1995). Other Cdk5 regulators in postmitotic neurons are the Cyclin family proteins, such as Cyclin D1, Cyclin E and Cyclin I (De Falco et al., 2004; Brinkkoetter et al., 2010; Odajima et al., 2011). In addition, glutathione-S-transferase P (GSTP1) binds to Cdk5 and modulates its activity in neurons (Sun et al., 2011). Furthermore, Cdk5 can be regulated by posttraslational modifications, such as phosphorylation and S-nitrosylation (reviewed in Shah and Lahiri, 2014). The N-terminal domain of the Cdk5 activator p35 is cleaved by the Ca^+^-dependent cysteine protease calpain, which generates p25, a C-terminal truncation peptide that contains all necessary elements for Cdk5 activation (Kusakawa et al., 2000; Lee et al., 2000; Nath et al., 2000; Dhavan and Tsai, 2001; Patzke et al., 2003). Since p25 is resistant to ubiquitin-mediated proteolysis, it substantially extends the activation period of Cdk5 (Patrick et al., 1999). Relevant to our studies, transient expression of p25 increased the number of synapses and dendritic spine density, enhancing hippocampal LTP and facilitating learning and memory (Fischer et al., 2005).

Notably, exposure to either amphetamine or cocaine increases the dendritic spine density in reward centers of the brain, suggesting that the molecular mechanisms involved in other forms of synaptic plasticity are hijacked by drugs of abuse (Berke and Hyman, 2000; Hyman and Malenka, 2001; Kolb et al., 2003; Robinson and Kolb, 2004). Although the dopaminergic system is the focus of intense research of motivational and reward-related learning (Li et al., 2003; Chen et al., 2010), the neurobiological basis of natural reward learning and memory cannot be fully explained without the contribution of the hippocampus, a brain area that sends a main output to the dopaminergic reward system. In addition, the hippocampus is a principal site of activity-dependent process during learning and memory (Grace et al., 2007). Little is known, however, about the effects of drugs of abuse on hippocampus.

Interestingly, recent reports have demonstrated that Cdk5 participates in addiction to drugs of abuse (Benavides and Bibb, 2004; Mlewski et al., 2008, 2016). The inhibition of Cdk5 activity with roscovitine decreases cocaine-induced dendritic spine formation in the nucleus accumbens, suggesting that Cdk5 is involved in cocaine-induced dendritic spine morphogenesis (Norrholm et al., 2003). Nevertheless, the detailed mechanism/s by which this or other signaling modules participate in dendritic spine formation induced by psychostimulants in the hippocampus or other brain areas remains to be determined.

In the present study, we demonstrate that amphetamine increases dendritic spine density in pyramidal neurons of the hippocampus. Using a two-amphetamine-injections protocol in a novel context, we observe an increase in dendritic spines density in hippocampal CA1 pyramidal neurons of young mice. In addition, amphetamine increases spine density in primary hippocampal cultures and organotypic slice cultures. Lastly, we provide evidence of the necessity of the Cdk5/p25 signaling complex and of calpain activity for amphetamine to increase spine density.

## Materials and Methods

### Animals

Mice harboring the thy1-green fluorescent protein (GFP) transgene (Feng et al., 2000) were generated by breeding male heterozygous B6.Cg-Tg(Thy1-EGFP)MJrs/J mice (Jackson Laboratory, Bar Harbor, ME, USA) with female C57BL/6J wild type mice. Animals were weaned at postnatal day (P) 21 and housed 4–5 per cage under a 12 h light/12 h dark cycle at constant temperature (22°C) with free access to food and water. All experiments were performed with P33–40 mice in an isolated behavioral room during the light cycle, between 10:00 AM and 3:00 PM. Embryonic Wistar rats were used for *in vitro* primary cultures. All animals used in the present study were born and reared at the vivarium of the INIMEC-CONICET-UNC (Cordoba, Argentina). All procedures and care performed in the animals were approved by National Department of Animal Care and Health (SENASA, Argentina) and were in accordance with National Institute of Health general guidelines for the Care and Use of Laboratory Animals. Best efforts were made to reduce the number of animals used and to minimize their suffering. The experimental protocol was reviewed and approved by Institutional Animal Care and Use Committee (CICUAL protocol No. 2016-0001) and complied with the regulations of the Guide for Care and Use of Laboratory Animals (National-Research-Council, 1996).

### Drugs

D-Amphetamine (Sigma-Aldrich, St. Louis, MO, USA and Parafarm, Buenos Aires, Argentina), N-Acetyl-L-leucyl-L-norleucinal (ALLN; Sigma-Aldrich, St. Louis, MO, USA) and roscovitine (Calbiochem, San Diego, CA, USA) were used in this study.

### Drug Administration

We performed the two-injection protocol of sensitization (TIPS), which consist of two phases: in the first day (phase 1) mice were treated with either an i.p. injection of 4 mg/kg amphetamine or an equivalent volume of saline solution (sal, 0.9% NaCl) and immediately placed in a novel context (open field) for 1 h. After 1 day without receiving any treatment, mice received either amphetamine or sal, and were placed for 1 h in the novel context (phase 2). Male mice were randomly divided into four treatment groups: (1) receiving two vehicle saline injections (sal/sal); (2) receiving saline first and then amphetamine injections (sal/amph); (3) receiving amphetamine first and then saline injections (amph/sal); and (4) receiving two amphetamine injections (amph/amph).

### Brain Fixation

Four hours after drug treatments, mice were anesthetized with an i.p. injection of chloral hydrate (0.1 mL/100 g) and transcardially perfused as described (Krapacher et al., 2010). The brains were cut coronally at 100 μm with a freezing microtome (Reicher-Jung Hn40, Leica Microsystems, Wetzlar, Germany) and the collected sections were mounted with Vectashield (Vector Laboratories, Burlingame, CA, USA).

### Primary Cultures of Dissociated Neurons

Cultures of dissociated hippocampal pyramidal cells from embryonic rats were prepared as described (Kunda et al., 2001). Neurons at 17–19 days *in vitro* (DIV) with long axons and well-developed dendritic arbors were used for all experiments.

### Organotypic Slice Cultures

Hippocampal slice cultures were prepared from P7 to P10 Wistar rats and maintained *in vitro* as described (Stoppini et al., 1991; Tyler and Pozzo-Miller, 2001). Briefly, rats were quickly decapitated and their brains aseptically dissected and immersed in ice-cold dissecting solution, consisting of Hanks’ Balanced Salt Solution (HBSS; ThermoFisher Scientific, Waltham, MA, USA) supplemented with glucose (41.55 mM) and antibiotics (1:100; penicillin/streptomycin). Hippocampi were then dissected and transversely sectioned into 400~500 μm slices using a custom-made tissue slicer (Katz, 1987) strung with 20 μm-thick tungsten wire (California Fine Wire Company, Grover Beach, CA, USA). Slices were incubated at 4°C for 30 min, and then plated on tissue culture inserts (0.4 μm pore size, Millicell-CM, Millipore Corporation, Billerica, MA, USA). Culture media contained Neurobasal-A (ThermoFisher Scientific, Waltham, MA, USA), heat-inactivated horse serum (20%) and L-glutamine (1 mM; ThermoFisher Scientific, Waltham, MA, USA). Organotypic cultures were maintained in incubators at 36°C, 5% CO_2_, 98% relative humidity. The concentration of horse serum in the culture medium was reduced from 20% to 10% at 4 DIV and again reduced to 5% 24 h later. After 24 h in medium containing 5% horse serum, slices were placed in serum-free medium (Neurobasal-A plus B-27 supplement, ThermoFisher Scientific, Waltham, MA, USA) until used for experiments.

### DNA Constructs

The following constructs were used: expression cDNA plasmid coding for enhanced yellow fluorescent protein (YFP) obtained from Clontech Inc (Mountain View, CA, USA); expression cDNA plasmid pcDNA3-dnCDK5 was generous gift of Dr. Philip W. Hinds; plasmid DNA coding for p35-full-length was generous gift of Dr. Christopher C. J. Miller. To generate the fusion protein of human p25 with GFP (p25-GFP), a DNA fragment encoding the p25 sequence was obtained by PCR using Pfu polymerase (Stratagene Cloning Systems, La Jolla, CA, USA), with a BamH1 restriction site introduced into the 5′-end and a SAL site into the 3′-end, and cloned into the pEGFP-N1 vector (Clontech Inc, Mountain View, CA, USA). The Cdk5 short hairpin RNA (shRNA) plasmid was constructed in pSilencer U6 1.0 vector (Ambion Inc, Austin, TX, USA). 5′-GGG AGATCTGTCTACTCAAAGAA and 5′-GGG ATT CTG TCA CAG CCG TAA CG were used as targeting sequences following the procedures described (Chuang et al., 2005). The DNA fragments containing U6-Cdk5-sh and U6-control-sh were inserted into pCAG-HcRed vector under the control of chick actin-minimal (CAG) promoter; the resulting plasmids were referred to as shCdk5. Western immunoblots of Cdk5-expressing N2a cells (brain neuroblastoma) confirmed that shCdk5 reduced Cdk5 protein levels (data not shown).

### Transfection Methods

For primary neuronal cultures, transient transfections were performed at 16 DIV on 30 mm dishes, using Lipofectamine 2000 (Invitrogen, San Diego, CA, USA) according to the manufacture’s recommendations. For experiments with organotypic cultures, slices were biolistically-transfected at 7 DIV, as described (Alonso et al., 2004). Briefly, prior to transfection, a mixture of penicillin/streptomycin/amphotericin B (ThermoFisher Scientific, Waltham, MA, USA) was added to the culture media in order to prevent contamination during biolistic transfection; this mixture was removed after 24 h. The plasmid (cDNAs of interest) were precipitated onto 25 mg of 1.6 μm-diameter colloidal gold at a ratio of 63.5 μg of YFP to 113.5 μg of interest plasmid and then coated onto Tefzel tubing. The success rate of co-transfection of the same neuron with two cDNA plasmids using the gene-gun has been demonstrated to be >90% (Boda et al., 2004; Moore et al., 2007), significantly reducing the likelihood of occurrence of YFP-positive and dnCDK5/p35/p25-negative neurons.

### Culture Fixation

Fifteen hours after transfection, hippocampal slice cultures were exposed to amphetamine (50 μM) and/or roscovitine (10 μM) for 48 h and then were fixed by immersion in 4% paraformaldehyde in 100 mM phosphate buffer (overnight at 4°C), and washed in PBS. Filter membranes around each slice were trimmed, and each slice was individually mounted on glass slides and cover slipped using Vectashield (Vector Laboratories, Burlingame, CA, USA). Cultured hippocampal neurons received the same treatment and then coverslips with neurons were fixed for 20 min in 4% paraformaldehyde in 100 mM phosphate buffer at room temperature and mounted on glass slides using FluorSave (Calbiochem^®^ Merck KGaA, Darmstadt, Germany).

### Laser-Scanning Confocal Microscopy

High-resolution images of tertiary and quaternary branches of apical dendrites of pyramidal neurons, displaying either GFP or YFP fluorescence throughout the whole dendritic tree, were acquired in Fluoview FV300 and FV1000 laser-scanning confocal microscopes (Olympus, Tokyo, Japan) using oil immersion 40× (NA 1.4) and 63× (NA 1.4) objectives (PlanApo, Olympus) and 3× digital zoom. GFP and YFP were excited using an Ar laser (488 nm), and detected using standard FITC filters. Series of optical sections were acquired in the *z*-axis at 0.12 μm intervals through individual apical dendritic branches.

### Quantitative Analyses of Dendritic Spine Density and Morphology

Small protrusions that extended ≤3 μm from the parent dendrite were identified as dendritic spine. Spines were counted in maximum-intensity projections of the z-stacks using ImageJ software (National Institute of Health, Bethesda, MD, USA), only if they appeared continuous with the parent dendrite. Spine density was calculated by quantifying the number of spines per dendritic segment, and normalized to 10 μm of dendrite length. The classification of morphological spine types was performed as described (Tyler and Pozzo-Miller, 2003). Spine types were grouped as immature-shaped thin (type-III) spines, mature-shaped spines, which included type-I (stubby) and type II (mushroom) spines, according published criteria (Boda et al., 2004). Images of pyramidal neurons in primary cultures were deconvolved in Fiji (Schindelin et al., 2012), followed by NeuronStudio processing in order to categorize dendritic spines into three morphological types based on their length and the dimensions of the spine head and neck: stubby, mushroom and thin (Rodriguez et al., 2008).

**Figure 1:**
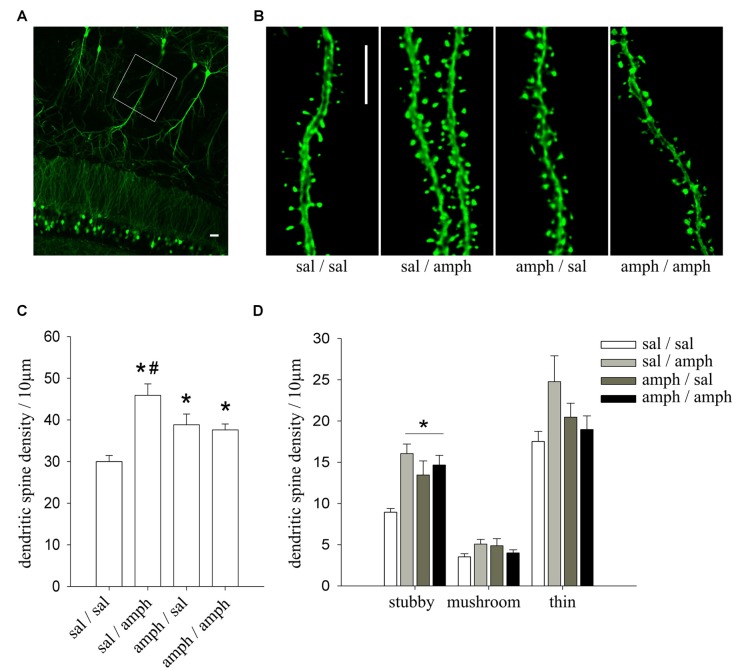
*In vivo* exposure to amphetamine increased dendritic spine density in hippocampal CA1 pyramidal neurons. **(A)** Representative image of the CA1 region of a thy1-green fluorescent protein (GFP) mouse showing GFP-expressing pyramidal neurons. White box indicates the region where dendritic segments were imaged at high magnification; scale bar = 20 μm. **(B)** Representative dendritic segments of CA1 pyramidal neurons of thy1-GFP mice; scale bar = 5 μm. **(C)** Dendritic spine density expressed as the number of spines normalized to 10 μm of dendritic length; data are mean ± SEM. *Indicates statistically significant differences vs. sal/sal, ^#^vs. all treatment groups (one way ANOVA, *p = 0.0001). **(D)** Density of the three different morphological spine types (stubby, mushroom and thin). *Indicates statistically significant differences (one way ANOVA, *p = 0.001). *N* = 4 mice; *n* = 3 dendritic segments per mouse.

**Figure 2:**
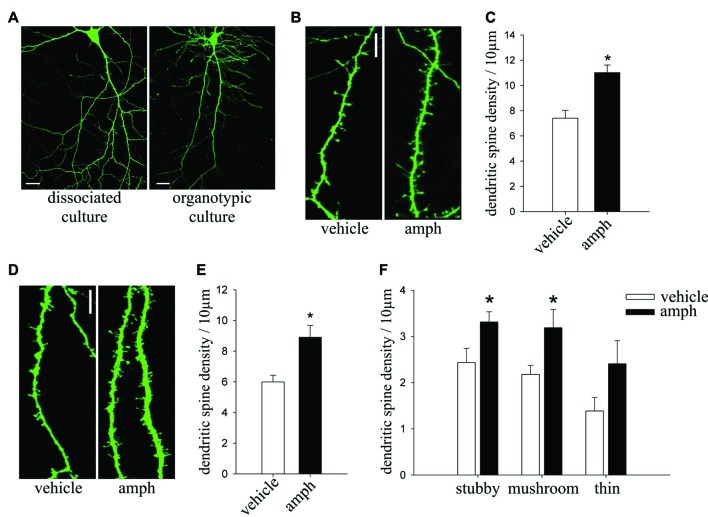
*In vitro* amphetamine exposure increased dendritic spine density in hippocampal neurons maintained in either dissociated or organotypic slice culture. **(A)** Representative images of hippocampal pyramidal neurons maintained in dissociated cultures or organotypic slice cultures (scale bar = 20 μm). **(B)** Representative examples of secondary or tertiary dendritic segments of cultured pyramidal neurons (17–19 DIV) expressing yellow fluorescent protein (YFP) and exposed to 50 μM amphetamine (amph) or vehicle for 48 h (scale bar = 5 μm). **(C)** Dendritic spine density (one-way ANOVA, *p = 0.001). **(D)** Representative maximum-intensity projections of confocal z-stacks of dendritic segments of YFP-expressing CA1 pyramidal neurons in slice culture (10 DIV), exposed to either 50 μM amphetamine or vehicle for 48 h (scale bar = 5 μm). **(E)** Dendritic spine density (one-way ANOVA, *p = 0.005). **(F)** Density of three different morphological spine types (stubby, mushroom and thin; one-way ANOVA, *p = 0.05). Samples of dissociated cultures (four different cultures): vehicle *n* = 10 neurons, amph *n* = 14. Samples of organotypic slice cultures (three different cultures): vehicle *n* = 13, amph *n* = 13.

**Figure 3:**
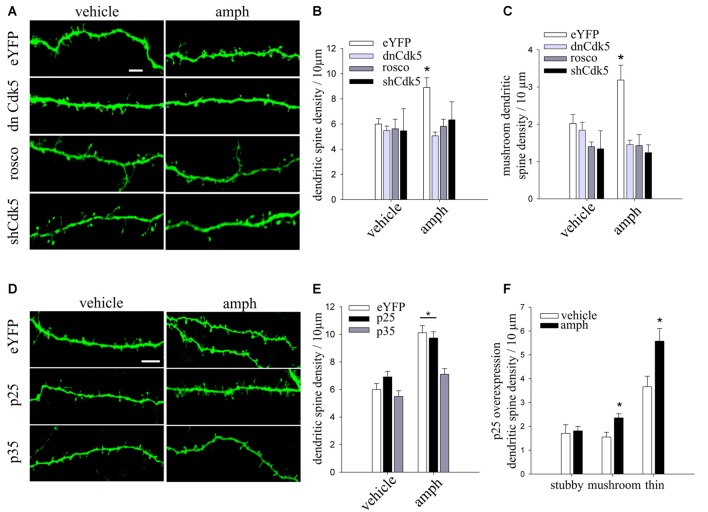
Cdk5 knockdown or pharmacological inhibition prevent amphetamine-evoked increase in dendritic spine density. **(A)** Representative maximum-intensity projections of confocal z-stacks of dendritic segments of YFP-expressing CA1 pyramidal neurons in slice culture (10 DIV), biolistically co-transfected with either dnCdk5 or a short hairpin RNA (shRNA) against Cdk5, and exposed to 50 μM amphetamine (amph), vehicle, or roscovitine (10 μM) for 48 h (scale bar = 5 μm). **(B)** Dendritic spine density in each condition (two-way ANOVA, *p = 0.01). **(C)** Density of mushroom spine type (two-way ANOVA, *p = 0.0001). **(D)** Representative maximum-intensity projections of confocal z-stacks of dendritic segments of CA1 pyramidal neurons in slice culture (10 DIV), biolistically co-transfected with either p35 or p25, and exposed to 50 μM amphetamine (amph) or vehicle for 48 h (scale bar = 5 μm). **(E)** Dendritic spine density for each condition (two-way ANOVA, *p = 0.005). **(F)** Density of three different morphological spine types (stubby, mushroom and thin; one-way ANOVA, *p = 0.01 and *p = 0.05 respectively). N = an average of nine neurons for each condition from three to eight slices of three different cultures.

**Figure 4:**
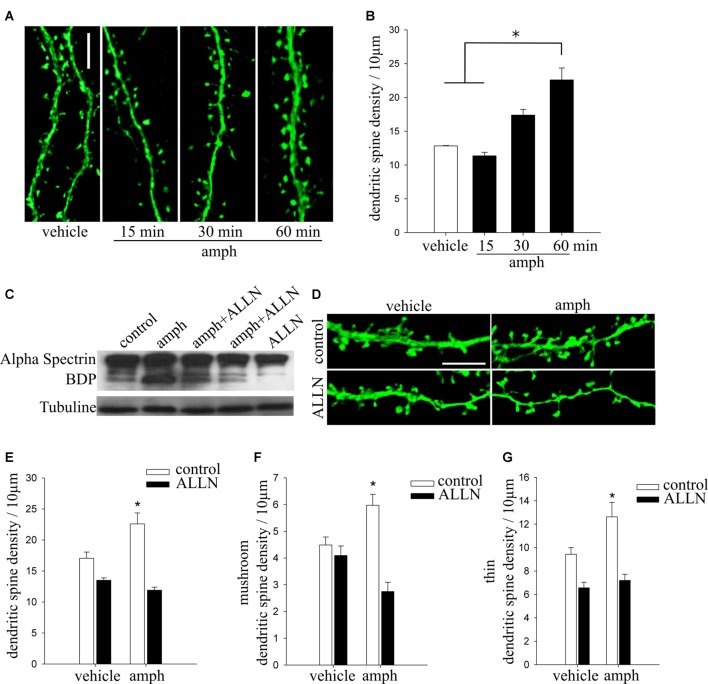
Calpain activity is necessary for amphetamine to increase dendritic spine density in hippocampal pyramidal neurons. **(A)** Representative dendritic segments of cultured pyramidal neurons (17–19 DIV) expressing YFP and exposed to either vehicle or amphetamine 50 μM (amph) for 15, 30 and 60 min (scale bar = 5 μm). **(B)** Dendritic spine density (one-way ANOVA, *p = 0.05). **(C)** Representative Western immunoblot showing that amphetamine increases calpain proteolytic activity. Total homogenate of hippocampal neuronal cultures (17–19 DIV) from control (line 1) or amphetamine-treated for 60 min (line 2) or pretreated with N-Acetyl-L-leucyl-L-norleucinal (ALLN; 10 μM) for 30 min and then exposed to amphetamine for 60 min (lines 3 and 4) or treated with ALLN alone as a control (line 5). **(D)** Representative maximum-intensity projections of confocal z-stacks of secondary or tertiary dendritic segments of cultured pyramidal neurons (17–19 DIV) expressing YFP and exposed to vehicle, 50 μM amph for 60 min, ALLN for 30 min followed by amph for 60 min, or ALLN alone (scale bar = 5 μm). **(E)** Dendritic spine density for all treatments (two way ANOVA, *p = 0.000001). **(F,G)** Density of mushroom (mush) **(F)** and thin **(G)** spine types (one way ANOVA, *p = 0.0001 and *p = 0.01, respectively).

The total length of secondary and tertiary apical dendrites analyzed for Figure 1 was: (i) sal/sal: 1244.58 μm from 20 dendritic segments of 9 cells; (ii) sal/amph: 1041.50 μm from 18 dendritic segments of 8 cells; (iii) amph/sal: 1078.29 μm from 20 dendritic segments of 8 cells; (iv) amph/amph: 1321.83 μm from 23 dendritic segments of 9 cells. For Figure 2, dissociated culture: (i) vehicle: 1432.03 μm from 34 dendritic segments of 10 cells; (ii) amph: 2084.79 μm from 40 dendritic segments of 14 cells; organotypic culture: (iii) vehicle: 840.93 μm from 26 dendritic segments of 12 cells; (iv) amph: 1204.28 μm from 34 dendritic segments of 15 cells. For Figure 3B: (i) eYFP-vehicle: 840.93 μm from 26 dendritic segments of 13 cells; (ii) dnCdk5-vehicle: 1528.945 μm from 34 dendritic segments of 14 cells; (iii) rosco-vehicle: 1227.58 μm from 16 dendritic segments of 6 cells; (iv) shCd5-vehicle: 689.22 μm from 18 dendritic segments of 3 cells; (v) eYFP-amph: 1204.28 μm from 34 dendritic segments of 12 cells; (vi) dnCdk5-amph: 1433.04 μm from 31 dendritic segments of 16 cells; (vii) rosco-amph: 1784.51 μm from 27 dendritic segments of 9 cells; (viii) shCd5-amph: 427.9 μm from 18 dendritic segments of 4 cells. For Figure 3E; (ix) eYFP-vehicle: 840.93 μm from 26 dendritic segments of 13 cells; (x) amph-vehicle: 1528.945 μm from 12 dendritic segments of 12 cells; (xi) p25-vehicle: 1241.19 μm from 26 dendritic segments of 7 cells; (xii) p25-amph: 1918.05 μm from 43 dendritic segments of 13 cells; (xiii) p35-vehicle: 581.81 μm from 13 dendritic segments of 8 cells; (xiv) p35-amph: 855.81 μm from 21 dendritic segments of 13 cells. For Figure 4B: (i) vehicle: 642.49 μm from 11 dendritic segments of 3 cells; (ii) 15 min amph: 717.48 μm from 11 dendritic segments of 3 cells; (iii) 30 min amph: 474.65 μm from six dendritic segments of 3 cells; (iv) 60 min amph: 749.2 μm from 20 dendritic segments of 4 cells. For Figure 4E; (v) vehicle-control: 792 μm from 19 dendritic segments of 7 cells; (vi) vehicle-ALLN: 304.6 μm from 8 dendritic segments of 4 cells; (vii) amph-control: 750 μm from 20 dendritic segments of 7 cells; (viii) amph-ALLN: 350.89 μm from 9 dendritic segments of 6 cells.

### Western Immunoblots

Crude synaptosomal fractions were obtained as described (Mlewski et al., 2008). All samples were valued for protein concentration (DC Protein Assay Kit, Bio-Rad Laboratory, Hercules, CA, USA) and equal protein amounts (25 μg) were separated into 10% sodium dodecyl sulfate-polyacrylamide gel electrophoresis and then transferred to Nitrocellulose membranes (Bio-Rad Laboratory, Hercules, CA, USA) as previously described (Krapacher et al., 2010). Membranes were incubated overnight at 4°C with their primary antibodies diluted in 1% skim milk in TBST. The antibodies used were anti-α-tubulin (1:3000, DM1A; Sigma-Aldrich, St. Louis, MO, USA) and anti-spectrin (1:1000, Millipore Corporation, Billerica, MA, USA). Membranes were then washed three times in TBST and incubated with a Licor secondary antibody (1:15,000, IRDye^®^ 800CW LI-COR Biosciences, Lincoln, NE, USA) or a horseradish peroxidase–conjugated antibody (1:2000; Jackson ImmunoResearch Laboratories, West Grove, PA, USA) for 1 h at room temperature. After two washes with TBST and two washes with TBS, bands were visualized using Odissey scanner (LI-COR Biosciences, Lincoln, NE, USA) or a chemiluminescence detection kit (ECL; Amersham Life Science, Buckinghamshire, England) according to the primary antibody used.

### Statistical Analyses

All data were analyzed using Statistica 7.0 (Statsoft, Inc., Tulsa, OK, USA). Statistical analyses were performed using ANOVAs. Significant main effects were further analyzed through Fisher’s LSD *post hoc* test (Fisher’s least significance difference test with a Type I error set at 0.05).

## Results

### *In Vivo* Administration of Amphetamine Increases Dendritic Spine Density in CA1 Hippocampal Pyramidal Neurons

We have previously shown that adolescent rats develop and express locomotor sensitization after an amphetamine two-injection protocol (4 mg/kg) when they were trained and evaluated in a novel environment (Mlewski et al., 2016). To test if this exposure to amphetamine affects dendritic spines in hippocampal neurons, we used the same protocol in male P35–40 thy1-GFP transgenic mice (line M), which express GFP in pyramidal neurons in a Golgi-type fashion (Feng et al., 2000; Figures 1A,B).

Amphetamine induced a significant increase of the density of spine in apical dendrites of CA1 pyramidal neurons in all groups (sal/amph, amph/sal and amph/amph) compared to sal/sal controls. ANOVA revealed a significant main effect of amphetamine (one-way ANOVA *F*_(3,30)_ = 10.304, *p < 0.0001), indicating that mice treated with amphetamine have higher dendritic spine density than sal/sal controls (Figure 1C). Amphetamine also modulated the morphology of individual spines, increasing the density of stubby spines (one-way ANOVA *F*_(3,30)_ = 7.3287, *p = 0.001; Figure 1D).

These results demonstrate that amphetamine, administrated in a two-injection protocol known to trigger locomotor sensitization, increases dendritic spine density in CA1 pyramidal neurons favoring the mature stubby type.

### *In Vitro* Administration of Amphetamine Increases Dendritic Spine Density in Hippocampal Pyramidal Neurons

To develop an *in vitro* assay amenable for testing molecular mechanisms, we exposed YFP-expressing cultured hippocampal neurons to amphetamine (50 μM) for 48 h (Figure 2A). Consistent with *in vivo* observations, amphetamine significantly increased spine density in YFP-expressing neurons maintained in primary culture (Figures 2B,C; one-way ANOVA *F*_(1,22)_ = 16.576, *p = 0.001). In addition, amphetamine (50 μM) induced a significant increase of spine density in YFP-expressing CA1 pyramidal neurons maintained in hippocampal slice cultures (Figures 2A,D,E; one way ANOVA *F*_(1,26)_ = 9.9457, *p = 0.005). Similar to its effects *in vivo*, amphetamine increased the density of mature stubby spines (one way ANOVA, *F*_(1,26)_ = 5.5401, *p = 0.05), and also of the mushroom type (one way ANOVA, *F*_(1,26)_ = 4.7140, *p = 0.05; Figure 2F).

Together with the observations *in vivo*, these results in two different *in vitro* preparations provide strong evidence of amphetamine-induced structural plasticity in pyramidal neurons of the hippocampus.

### Cdk5 Activity Is Necessary for Amphetamine to Increase Dendritic Spine Density in Hippocampal Pyramidal Neurons

Considering that Cdk5 plays a role in cocaine-induced dendritic spine formation in the *nucleus accumbens* (Norrholm et al., 2003), we tested whether it is also involved in the effects of amphetamine on CA1 hippocampal pyramidal neurons. To this end, we used three strategies: (1) slice cultures were biolistically co-transfected with YFP and dominant negative Cdk5 (dnCdk5), which abolishes endogenous Cdk5 activation by p35/p25 (Tsai et al., 1994); (2) pharmacological inhibition of Cdk5 kinase activity with the specific inhibitor roscovitine; and (3) slice cultures were biolistically co-transfected with YFP and a Cdk5 short harping interfering RNA (shCdk5) to suppress Cdk5 expression.

Expression of dnCdk5, shRNA-mediated Cdk5 knockdown, and pharmacological Cdk5 inhibition with roscovitine all prevented the increase in spine density induced by amphetamine (Figures 3A,B). In addition, all three Cdk5 manipulations blocked the increase in the density of mushroom spines induced by amphetamine (two-way ANOVA *F*_(3,68)_ = 7.47556 *p = 0.0001; Figure 3C). None of these Cdk5 manipulations by themselves affected dendritic spine density or morphology (two-way ANOVA *F*_(3,68)_ = 3.6015, *p = 0.01; Figures 3A,B). These results demonstrate that amphetamine’s effects on dendritic spines require intact Cdk5 activity.

Cdk5 activity is induced by the interaction with a regulatory subunit, either p35 or p25 (proteolytic fragment of p35; Tsai et al., 1994; Tang et al., 1995). In addition, the level of p35 is the rate-limiting factor for the activity of Cdk5 (Takahashi et al., 2005). To examine the role of p35 and p25 in the amphetamine effect on spine density, CA1 pyramidal neurons in slice cultures were biolistically co-transfected with YFP and either p35 or p25. Overexpression of p35 prevented the increase in spine density induced by amphetamine. In contrast, p25-expressing neurons exposed to amphetamine showed the typical increase in spine density (Figures 3D,E; two-way ANOVA *F*_(2,60)_ = 3.8051 *p = 0.005). In addition, p25-expressing neurons exposed to amphetamine showed higher densities of mature mushroom spine as well as immature thin spines (Figure 3F; one-way ANOVA, *F*_(1,18)_ = 8.1234 *p = 0.01 and *F*_(1,18)_ = 5.7472 *p = 0.05 respectively).

Together, these results demonstrate that amphetamine’s effect on spine density requires proper levels of p35 to regulate Cdk5 activity, and that overexpression of p25 does not affect the increase of dendritic spine density induced by amphetamine.

### Calpain Activity Is Necessary for Amphetamine-Induced Increase Dendritic Spine Density in Hippocampal Pyramidal Neurons

Proteolytic cleavage of p35 by the Ca^2+^ activated cysteine protease calpain (Liu X. et al., 2008) results in the formation of p25, which causes hyperactivation of Cdk5 (Kusakawa et al., 2000; Lee et al., 2000). Considering that the only way to generate p25 is by proteolytic cleavage of p35 protein by calpain (Kusakawa et al., 2000), and that amphetamine increases intracellular Ca^2+^ levels (Licata and Pierce, 2003; Gnegy et al., 2004; Mills et al., 2007), we tested if calpain activity is involved in amphetamine’s effects on dendritic spine density in hippocampal neurons.

We first tested if exposure to amphetamine activates calpain activity. Cell lysates from hippocampal neurons were prepared 60 min after application of amphetamine (50 μM) because that is when its effect on dendritic spine density becomes statistically significant (Figures 4A,B; one way ANOVA, *F*_(3,24)_ = 3.5324 *p = 0.05). Calpain activity was examined by Western immunoblots using an antibody that recognizes well-characterized calpain-specific α-spectrin breakdown products (α-spectrin-BDP; Zhang et al., 2009; Ma et al., 2012). A 60 min exposure to amphetamine induced robust calpain activity (Figure 4C, line 2), an effect blocked by preincubation with the calpain inhibitor ALLN (Figure 4C, lines 3, 4).

Finally, we tested if inhibition of calpain activity prevents amphetamine’s effect on spine density. Indeed, the calpain inhibitor ALLN abolished the increase in spine density observed 60 min after exposure to amphetamine (Figures 4D,E; two way ANOVA, *F*_(3,57)_ = 12.18 *p = 0.000001). Calpain inhibition also prevented the amphetamine-induced increase in the densities of mushroom and thin spines types (Figures 4F,G; one way ANOVA, *F*_(3,57)_ = 11.021 *p = 0.0001 and *F*_(3,57)_ = 7.9454 *p = 0.01, respectively).

Taken altogether, our findings demonstrate that amphetamine increases dendritic spine density in hippocampal neurons by triggering the proteolytic cleavage of p35 by calpain, generating p25 to activate Cdk5.

## Discussion

This study provides strong findings and novel insight into the molecular bases by which amphetamine increases dendritic spine density in hippocampal neurons. Using thy1-GFP transgenic mice, we show that amphetamine administrated in a two-injection protocol increased dendritic spine density in CA1 pyramidal neurons and shifted the density of morphological types in favor of mature stubby spines. Using two different *in vitro* systems, we also show this effect of amphetamine requires proteolytic cleavage of p35 by calpain to generate p25 that in turn activates Cdk5.

A great number of studies into the pathophysiology of drug abuse have concentrated on the dopaminergic reward system, valuing the neural changes induced during addiction, relapse, and abstinence in this natural reward regions (Chen et al., 2010; Deadwyler, 2010; Collo et al., 2014). Even though the dopaminergic system is central of intense research of motivational and reward-related learning, the neurobiological basis of reward learning and memory are uncompleted without the contribution of the hippocampus, which sends a main output to the reward system. Considering that this brain structure lies upstream of the striatal dopaminergic circuit, synaptic plasticity within the hippocampus may alter the transmission of information throughout the brain’s reward system (Grace et al., 2007). Several studies exhibit that psychostimulant drugs produce persistent alterations in other forebrain regions as well, in particular, in the orbital frontal cortex (Fein et al., 2002; Franklin et al., 2002; Paulus et al., 2002; Adinoff et al., 2003; Bolla et al., 2003; Matochik et al., 2003). Moreover, human psychostimulants users, as well as cocaine-exposed monkeys, show deficits in the execution of cognitive tasks (Rogers et al., 1999; Ornstein et al., 2000; Jentsch et al., 2002). Also, studies in rats have shown that chronic cocaine-treatment produce deficits in discrimination learning (Schoenbaum et al., 2004). Together, these studies indicate that psychostimulant drugs also produce lasting alterations in other brain areas related with higher-order associative learning, in addition to changes in reward circuit. However, little is known, about the cellular and molecular mechanisms of the effects of psychostimulant drugs in the hippocampus. In the few studies performed in rodents, psychostimulant-induced structural plasticity in non-reward regions like the hippocampus, has been studied using long chronic administration regimens (Rademacher et al., 2006; Shen et al., 2006; Boikess et al., 2010; Ahn et al., 2013). Here, we show for the first time that amphetamine administrated in a two-injection protocol increases dendritic spine density in CA1 pyramidal neurons of thy1-GFP transgenic mice. Interestingly, the largest effect was observed in mice that had the first experience with the drug (group sal-amph or amph/sal), suggesting that the first exposure to amphetamine is enough to induce structural plasticity in the hippocampus. Amphetamine also modulated the morphology of individual spines by increasing the density of stubby spines. This type of spines is the most abundant during development and is considered very plastic structures, strongly coupled to the dendritic shaft (Noguchi et al., 2005; Schmidt and Eilers, 2009). Interestingly, similar to its effects *in vivo*, amphetamine increased the density of stubby spines and also mushroom type *in vitro* condition. Taken together, these data provide strong evidences of amphetamine-induced structural plasticity in hippocampal pyramidal neurons, both *in vivo* and *in vitro* conditions. The particular morphology of a dendritic spine may play an important role in determining its function, a concept that has been extensively reviewed (Shepherd, 1996; Yuste and Majewska, 2001; Nimchinsky et al., 2002). Two morphological features of dendritic spines seem to be critical for their role as biochemical compartments: the geometrical dimensions of the head and neck, which affect the diffusion and/or accumulation and retention of ions and molecules generated by synaptic signaling at the postsynaptic density (e.g., free ionized Ca^2+^, activated CaMKII). In addition, the morphology and size of spines correlate with the strength of the synapses they bear (Pierce and Lewin, 1994). The spine head volume is directly proportional to the number of docked vesicles at the active zone of presynaptic terminals (Harris and Sultan, 1995; Boyer et al., 1998; Schikorski and Stevens, 1999) and to the number of AMPARs (Nusser et al., 1998). Moreover, the expression of AMPARs within individual spines of different morphologies was mapped by 2-photon uncaging of MNI-glutamate, revealing that mushroom (Type-II) spines show larger AMPAR-mediated responses than thin (Type-III) spines (Matsuzaki et al., 2001). These studies provide functional evidence that larger spines represent stronger synapses, as defined by their expression of AMPARs (Kasai et al., 2003). The induction of LTP leads to an increase in spine head size, while long-term depression (LTD) causes spine shrinkage and retraction, which correlates well with the synaptic levels of AMPARs observed after the induction of such bi-directional synaptic plasticity. Despite the large literature, it remains unclear to what extent the plasticity of dendritic spines drives the pathological behavior evident in psychostimulants addiction.

Previously, we demonstrated that both acute and chronic amphetamine treatment *in vivo* induced a significant increase in Cdk5 activity (Mlewski et al., 2008). Other studies explored the relationship between the activity of Cdk5 and dendritic spine maintenance (Fu et al., 2007; Lai et al., 2012). Here, we show that the expression and activity of Cdk5 is necessary for amphetamine to modulate dendritic spine density and their morphology: shRNA-mediated Cdk5 knockdown, pharmacological inhibition with roscovitine, and suppression of endogenous Cdk5 activity with a dominant negative mutant, all completely abolished the amphetamine-induced increase in spine density in hippocampal CA1 pyramidal neurons maintained in slice culture. These results are consistent with previous reports of reduction in cocaine-induced increase in spine density in the *nucleus accumbens* by roscovitine (Norrholm et al., 2003). In addition, dendritic spine density is lower in hippocampal neurons of the double inducible-p35cKO and p39KO mice, indicating that Cdk5 activity is necessary for the maintenance of the density of dendritic spine, at least in hippocampal neurons in adult naïve mice (Mita et al., 2016).

Considering that Cdk5 and its activators p35 and p25 have been involved in the effects of chronic exposures to cocaine (Bibb et al., 2001), methamphetamine (Chen and Chen, 2005), and amphetamine (Mlewski et al., 2008), we further investigated the role of p35 and p25 in amphetamine’s effect on dendritic spine density in hippocampal CA1 pyramidal neurons. Overexpression of p25 did not affect the amphetamine-evoked increase in spine density. This result is in line with the increase in dendritic spine density and number of synapses in an inducible transgenic mouse that transiently overexpress p25 in the hippocampus (Fischer et al., 2005). In contrast, overexpression of p35 completely abolished the effect of amphetamine on dendritic spine density. Consistent with this result, it has been demonstrated that high p35 levels binds microtubules through its N- terminal domain and removes it from Cdk5, rendering the kinase inactive (Shah and Lahiri, 2014). In addition, p35 binding to microtubules promotes microtubule bundling (He et al., 2008) and their stabilization (Hou et al., 2007). Moreover, p35 phosphorylation at Ser-8 and Thr-138 sites by Cdk5 suppresses its calpain-catalyzed truncation, but increases its F-actin bundling and binding activities, which in turn would stabilize actin filaments (Kamei et al., 2007; He et al., 2011).

We previously demonstrated a transient increase of p25 expression in the synaptosomal fraction of rat striatum after acute amphetamine treatment (Mlewski et al., 2008). These enhanced p25 levels were associated with a significant rise in Cdk5 activity and an enrichment of p21-activated kinase 1 (PAK1) phosphorylated on Thr-212, the specific site for Cdk5 kinase (Mlewski et al., 2008). PAK1 is involved in actin remodeling, dendritic spine morphogenesis, excitatory synapse formation, and synaptic plasticity (Zhang, 2005). It has been suggested that Cdk5 allows PAK1 to switch from an active to an inactive state and thereby mediate a rapid and dynamic regulation of the actin cytoskeleton (Rashid et al., 2001), thus facilitating dendritic spine formation. Altogether, these observations support the model that p25-regulated Cdk5 activity is specifically involved in cellular events underlying psychostimulant-induced synaptic plasticity.

Considering that calpain-mediated cleavage of p35 is the only way to generate p25 (Kusakawa et al., 2000; Kerokoski et al., 2004; Zhu et al., 2011), and that cocaine and amphetamine increase intracellular Ca^2+^ levels, we next tested whether the amphetamine effect on dendritic spine density in hippocampal neurons requires calpain activity. Calpains are a family of cysteine, non-lysosomal, calcium-dependent proteases whose substrates are involved in signal transduction, cytoskeleton remodeling, cell differentiation, vesicular trafficking, axonal degeneration and neuronal death (Liu J. et al., 2008). A unique feature of this protease is that its processed substrates often acquire different functions (Ma, 2013). An example of this is p35, which is cleaved to p25 by calpain. The resultant Cdk5/p25 complex exerts several functions associated to corticogenesis, cytoskeleton dynamic, synaptic plasticity, and dendritic spine formation (Kerokoski et al., 2004; Fischer et al., 2005; Mlewski et al., 2008; Barnett and Bibb, 2011; Lai et al., 2012). Considering that Ca^2+^-dependent processes are fast and transient, we first determined the time course of amphetamine’s effect on dendritic spine density in hippocampal cultures. Sixty minutes of amphetamine exposure were sufficient to significantly increase dendritic spine density, suggesting rapid post-translational modification of specific proteins, such as calpain and its substrates. Consistently, strong calpain proteolytic activity was detected in amphetamine-treated cultures, and pharmacological inhibition prevented the increase in dendritic spine density in hippocampal CA1 pyramidal neurons exposed to amphetamine. In line with these observations, conditional deletion of calpain-1 and calpain-2 results in lower dendritic spine density in the apical and basal dendrites of hippocampal CA1 pyramidal neurons (Amini et al., 2013). Interestingly, calpain inhibition also prevented the amphetamine-induced increase in the densities of mushroom and thin spines types, precisely the same dendritic spines types that are increased by amphetamine in p25-overexpressing neurons. Initially it was thought that spine synapses originate from established synapses first on the dendritic shaft by the enlargement and then by narrowing of the neck and the emergence of the spine head (Miller and Peters, 1981). Otherwise, filopodia have been considered as precursors of mature dendritic spines. Considering that most of spines have a presynaptic partner (Gray, 1959; Harris and Kater, 1994), it has been suggested that dendritic filopodia search for presynaptic axon terminals to form synapses, giving rise to more developed spines after the first contact (Jontes and Smith, 2000; Yuste and Bonhoeffer, 2004). Furthermore, time-lapse experiments in hippocampal slices maintained in culture, subsequently demonstrated that filopodia are highly dynamic structures, extending and retracting in short time periods (Dailey and Smith, 1996). Besides, it has been shown that filopodia from cultured neurons establish contact with axons, and its number decreased in correlation with the increase in spines number (Ziv and Smith, 1996). Thus, the initial contact with axons triggers the formation of spines from filopodia. Finally, *in vivo* imaging of dendritic spines in the neocortex of developing and adult animals from multiphoton microscopy supports the fact that spines constantly form by seeking out presynaptic partners in the surrounding neuropil and stabilizing into functional spines of varied morphology, a process driven by sensory experience (Lendvai et al., 2000; Trachtenberg et al., 2002; Holtmaat et al., 2005; Knott et al., 2006).

In summary, we demonstrate that amphetamine increases dendritic spine density in hippocampal neurons through the proteolytic cleavage of p35 into p25 by calpain, which in turn results in p25-depedent activation of Cdk5. p25-activated Cdk5 likely endows different properties to Cdk5, resulting in different roles at discrete levels of organization and different regulatory strategies in the recognition of particular substrates. The apparent lack of p25 in naïve conditions, together with its ability to enhance synaptic plasticity, suggests that it is shaped only during particular events, such as during learning (Fischer et al., 2005) or exposure to drugs of abuse (Mlewski et al., 2008). It is possible that in turn, the Cdk5/p25 complex could modulate critical substrates involved in the reorganization and dynamics of the actin cytoskeleton (Dietz et al., 2012), a fundamental mechanism in the formation of dendritic spines and the excitatory synapses they are part of. Revealing the cellular and synaptic mechanisms underlying drug addiction and the identification of the molecules involved will yield novel opportunities of potential therapeutic targets for the treatment of addictive disorders.

## Author Contributions

All authors were responsible for the design of the study. SF, GF, VD and MVP performed experimental procedures and collected data. ECM, SF and CA conducted statistical data analysis. LM and CAC performed cell culture and FAK conducted biochemical assays. The findings were interpreted and the manuscript was drafted by SF, GF, LP-M, DHM and MGP. All authors critically reviewed content and approved the final version for publication.

## Conflict of Interest Statement

The authors declare that the research was conducted in the absence of any commercial or financial relationships that could be construed as a potential conflict of interest.
